# Assemblage of the Egg Parasitoids of the Invasive Stink Bug *Halyomorpha halys*: Insights on Plant Host Associations

**DOI:** 10.3390/insects11090588

**Published:** 2020-09-01

**Authors:** Livia Zapponi, Marie Claude Bon, Jalal Melhem Fouani, Gianfranco Anfora, Silvia Schmidt, Martina Falagiarda

**Affiliations:** 1 Research and Innovation Center, Fondazione Edmund Mach, 38010 San Michele all’Adige, TN, Italy; jalal.fouani@fmach.it (J.M.F.); gianfranco.anfora@fmach.it (G.A.); 2 USDA-ARS European Biological Control Laboratory, 810 Avenue du Campus Agropolis, 34980 Montferrier le Lez, France; mcbon@ars-ebcl.org; 3 Center for Agriculture, Food and Environment (C3A), University of Trento, 38010 San Michele all’Adige, TN, Italy; 4 Entomology Group, Institute for Plant Health, Laimburg Research Centre, Laimburg 6, 39040 Auer (Ora), South Tyrol, Italy; Silvia.Schmidt@laimburg.it (S.S.); Martina.Falagiarda@laimburg.it (M.F.)

**Keywords:** biological control, BMSB, haplotype, host-parasitoid association, invasive insect, *Trissolcus*

## Abstract

**Simple Summary:**

The Brown Marmorated Stink Bug (BMSB), *Halyomorpha halys*, is an invasive polyphagous species that causes extensive damage to fruits and vegetable crops. Available chemical treatments and mechanical measures are often insufficient to keep this pest under control. In the last decade, the scientific community has made several efforts in order to identify and select suitable natural enemies to use in biological control program. Several native and exotic parasitoid species attacking *H. halys* eggs have been registered in the invaded countries. In this study, the distribution of these parasitoids was examined in Trentino-Alto Adige, in Northern Italy, by combining two different methods. The research revealed the presence of seven parasitoid and one hyperparasitoid species, which are present in different contexts and on several host plants. The most abundant species, *Anastatus bifasciatus* and *Trissolcus mitsukurii*, showed different patterns in distribution, with a higher presence of *A. bifasciatus* in urban areas and *Tr. mitsukurii* in orchards. Our results proved that *H. halys* natural enemies are adapted to the environmental conditions of the study area. The gathered data on the plant host-associations will support the development of future biological control programs.

**Abstract:**

*Halyomorpha halys* (Stål) (Hemiptera: Pentatomidae) is an invasive alien species and a key agricultural pest. Its native parasitoids (*Trissolcus japonicus* Ashmead and *Tr. mitsukurii* Ashmead) have been registered in several countries where *H. halys* brought dramatic economic losses and where biological control is considered to be the most effective long-term solution. By searching for stink bug egg masses and exposing sentinel egg masses, we monitored the distribution of native and exotic egg parasitoids in Trentino-Alto Adige (Italy), an area where both the host and parasitoids are in expansion. We recorded ten pentatomids, seven parasitoid species, with the first report of *Tr. japonicus* in this area and a hyperparasitoid. In the assemblage, *Anastatus bifasciatus* (Geoffroy) and *Tr. mitsukurii* were the dominant parasitoids, with a different distribution in terms of context and host plants. Sycamore was the host plant where the highest number of naturally laid parasitized egg masses (26%) were recorded. *Trissolcus mitsukurii* showed the highest parasitism rate, and was often found in apple orchards. The emergence of exotic parasitoids showed a temporal delay compared to native ones. Sequence analysis of 823 bp of the *CO1* mitochondrial gene revealed that the recovered *Tr. japonicus* and *Tr. mitsukurii* harbored one single haplotype each. These haplotypes were previously found in 2018 in Northern Italy. While sentinel egg masses proved to be very effective in tracking the arrival of exotic *Trissolcus* species, the collection of stink bug egg masses provided fundamental data on the plant host species. The results lend strong support to the adaptation of exotic *Trissolcus* species to the environmental conditions of the range of introduction, providing new information on plant host-associations, fundamental for the development of biological control programs.

## 1. Introduction

*Halyomorpha halys* Stål (Hemiptera: Pentatomidae), the brown marmorated stink bug, is a highly polyphagous invasive species. Since its first detection in the United States in 1996 [1], it has been found in several European countries, including Italy [2] and Switzerland [3], in Canada [4] and in South America [5]. Its invasiveness is ensured by high dispersal capabilities [6] and its hitchhiker behavior [7]. *Halyomorpha halys* appears to have over 100 host plants [8]. Its strong-flying capacity allows the adults to escape when pesticides are applied [9], making the effectiveness of chemical control uncertain. In addition, as more environmentally friendly control measures are urgently needed in Europe, an alternative is to rely on classical biological control that may last in the long-term, due to the self-sustaining ability of biocontrol agents [10].

*Halyomorpha halys’* egg parasitoids have been subject to many studies over the years. While the recorded rates of emergence of native parasitoids in Europe from *H. halys* eggs were low [11], in China, Zhang et al. [12] found that *Trissolcus japonicus* Ashmead (Hymenoptera: Scelionidae) was the most common and effective in terms of *H. halys* control, due to its ability to recognize specific volatiles from the egg masses. Another egg parasitoid of *H. halys*, *Trissolcus mitsukurii* Ashmead, was found to be predominant in Japan [13,14]. Adventive populations of *Tr. japonicus* were first discovered from sentinel egg masses in Maryland (US) in 2014 [15], then in Switzerland in 2017 [16]. In 2018, both *Tr. japonicus* and *Tr. mitsukurii* were found in Northern Italy [16]. The natural expansion of these egg parasitoids may represent the starting point for the implementation of classical biological control programs in Europe.

The monitoring of the abundance of *H. halys* egg parasitoids is commonly based on (a) exposure of sentinel egg masses [15,17], (b) field collection of *H. halys* egg masses [18,19], and c) the use of yellow sticky traps [20,21]. All of these methods present their advantages and drawbacks in terms of the time required to perform the different survey phases. Although the use of sentinel egg masses depends upon a fairly lengthy process of rearing *H. halys* specimens for the production of egg masses, this approach allows monitoring effectively the presence of parasitoids, when and where the availability of naturally laid egg masses is limited. Conversely, the collection of naturally laid egg masses does not require preparation time but needs more training, and the search can be time demanding. However, these methods represent the best approaches to gather fundamental data on species which ecology has not been studied in detail.

While previous experiments have focused on the effect of plant volatiles in laboratory conditions (e.g., [22,23]), the effect of plant stimuli and landscape factors on *H. halys* natural enemy community is a topic that needs further investigation [24]. The present study, by combining the collection of naturally laid egg masses and the exposure of sentinel egg masses, aims at examining the presence and distribution of native and exotic egg parasitoids in Trentino-Alto Adige (Northern Italy). In this area of the recent arrival of *H. halys* exotic parasitoids, we also investigated the possible effect of plant hosts on the parasitization of stink bug eggs by different parasitoid species. We examined whether the landscape context (urban or agricultural) affected the composition of the parasitoid assemblages. Furthermore, we analyzed the rates of parasitism and phenology of exotic and native stink bug parasitoid species to gather fundamental data on host-parasitoid interactions. Finally, we examined the contribution of plant species in the parasitoid-host location to identify potential sites for both monitoring the targeted parasitoid species and developing future classical and conservation biological control programs in Italy.

## 2. Material and methods

### 2.1. The Search for Naturally Laid Stink Bug Egg Masses

The monitoring of naturally laid egg masses was carried out in 2019, from June until October, in the Trentino-Alto Adige Region (Figure S1). It was part of a large-field survey conducted in North-Central Italy, promoted by the Council for Agricultural Research and Economics–Research Centre for Plant Protection and Certification (Florence). Field surveys were performed each week, where egg masses of *H. halys* and other stink bug species were collected. Several types of habitats were taken into account, associated to cultivated fields and urban areas. In each site, egg masses were searched on the underside of leaves, examining the canopy of trees and shrubs and herbaceous plants. Plant host species were not chosen *a priori*, in order to obtain a more complete overview of the hosts used in the study area. For each collected egg mass, the location and plant species were recorded. A code was assigned to each egg mass, and they were transferred to the laboratory for identification.

### 2.2. Rearing of H. halys

Egg masses of *H. halys* were produced in two facilities, i.e., the Edmund Mach Foundation (S. Michele all’Adige, TN) and the Laimburg Research Center (Laimburg, BZ) where rearing conditions were similar. The colonies were kept in plastic mesh cages (30 × 30 × 30 cm) at 25 ± 1 °C, 60–70% relative humidity (RH) and 16 L:8 D light cycle. The specimens were provided twice a week with fresh vegetables, fruit, and a mixture of seeds. In each cage, white fabric was provided as a support for the oviposition and egg masses were collected daily to ensure their freshness (24 h), and either exposed directly or stored in a −20 °C freezer.

### 2.3. Sentinel Egg Masses Exposure with Cages

Since *Ailanthus altissima* (Mill.) Swingle is considered a highly attractive species for *H. halys* [17], we selected 11 sites with *Ai. altissima* trees bordering apple orchards and vineyards (Figure S1). In each site, we selected two trees, at least 10 m apart from one another, which were used for the whole duration of the experiment. Cylindrical containers (20 cm × 11 cm) made of metal diamond mesh (4 mm × 4 mm) were specifically designed in order to (a) allow the entrance of parasitoids and prevent egg predation, (b) offer support for vials filled with water, used to keep the leaves fresh; (c) attach ropes to suspend the cages in the canopy of the trees. We used two egg masses per cage—a fresh and frozen egg mass were attached with a stapler, using the underlying piece of fabric, to the underside of the fresh *Ai. altissima* leaves (removed from the trees). Each egg mass was composed of 20 to 30 eggs. The egg masses in the cages were exposed for four days every two weeks, from May to August 2019.

### 2.4. Sentinel Egg Masses Direct Exposure

To evaluate the impact of predation and to test the efficacy of a less time-consuming method, we also exposed egg masses directly attached to the underside of leaves. We considered several tree and shrub species, where the presence of *H. halys* adult and nymphal stages were detected: *Acer campestre* L., *Ac. negundo* L., *Ac. pseudoplatanus* L., *Ai. altissima*, *Cornus mas* L., *Diospyros kaki* L.f., *Fraxinus excelsior* L., *Prunus padus* L., *P. persica* (L.) Batsch, *P. spinosa* L., *Sorbus aria* (L.) Crantz and *Tilia platyphyllos* Scop. The selected branch was marked with a colored label to help the recovery of the egg masses. Fresh and frozen egg masses were exposed for four days every two weeks, from July to September 2019. Each egg mass was composed of 20 to 30 eggs.

### 2.5. Egg Mass Evaluation

After recovery, all the egg masses (naturally laid and sentinel) were kept in plastic Petri dishes in growth chambers set at 25 ± 1 °C, 60–70% RH, and 16L: 8D light cycle until parasitoids or nymphs emerged (for a maximum of 45 days). The number of hatched stink bugs and parasitoids was recorded, and parasitoids were conserved in 95% ethanol for identification and genetic analyses. If parasitoid emergence took place in the field before collection, eggs were classified as parasitized. Exit holes were categorized according to Jones et al. [25] and Sabbatini Peverieri et al. [26] with a stereomicroscope. Unhatched eggs were also counted.

Two indices were used to compare the efficacy of the sentinel egg mass surveys: the discovery efficiency (the percentage of egg masses with at least a parasitized egg), and the exploitation efficiency (the number of parasitized eggs over the total number of eggs in the egg mass) [27,28]. The latter index was also applied to field-collected egg masses to compare the efficacy of the emerged parasitoids.

### 2.6. Parasitoid Identification

For the identification of *Trissolcus* species, the following revisions and keys were used: Talamas et al. [29,30] and Tortorici et al. [31]. The determination of the *Telenomus* individuals was performed using the classification key by Johnson [32]. *Anastatus bifasciatus* Geoffroy was identified according to Askew and Nieves-Aldrey [33]. The identification of the emerged parasitoids was supported by Dr. Francesco Tortorici (University of Turin, Italy).

### 2.7. mtDNA Haplotype Analysis

Specimens of *Tr. japonicus* and *Tr. mitsukurii*, collected alive and fixed in 96% ethanol, were used for nondestructive DNA extraction using the Qiagen DNeasy kit^®^ (Qiagen, Hilden, Germany) following the protocol published in Sabbatini Peverieri et al. [26]. A comprehensive list of all specimens sampled with author and year, host plant, and locality data is given in Table S1 in the Supplementary Materials, and all the voucher specimens are archived at the Fondazione Edmund Mach in Italy. An approximately 850 bp fragment of the 5′ region of the mitochondrial Cytochrome Oxidase Subunit I (*COI*) was amplified using the primers LCO1490puc [34] and C1-N-2353 [35]. PCR was performed in a 30 µl total volume with 2 µl of DNA template, 0.2 mM of each dNTP, 0.3 µM of each primer, 1× CoralLoad PCR Buffer (including 15 mM of MgCl_2_), and one Unit of *Taq* DNA Polymerase (Qiagen, Hilden, Germany). Thermocycling conditions were (1) initial denaturation at 94 °C for 5 min, (2) 35 cycles of denaturation at 94 °C for 60 s, annealing at 50 °C for 30 s, extension at 72 °C for 60 s, (3) a final elongation step of 7 min at 72 °C. PCR products were bidirectionally sequenced and sequences were assembled and edited as described in Ganjisaffar et al. [36]. All residual DNAs were archived at European Biological Control Laboratory (EBCL). To confirm the taxonomy of all the morphologically identified specimens, the *CO1* sequences obtained were compared with sequences present in Genbank by similarity search using the Basic Local Alignment Search Tool (http://ncbi.nlm.nih.gov/Blastn). To identify the haplotypes, sequences of both species were aligned using Clustal W, as implemented in MEGA X [37] with the dataset of 617 bp barcode haplotypes of *Tr. mitsukurii* and *Tr. japonicus* published by Sabbatini Peverieri et al. [26].

### 2.8. Statistical Analysis

All the statistical and descriptive analyses were performed with R v. 3.5.2 (R Core Team 2018, Vienna, Austria). The influence of plant host species on parasitization (yes/no) was evaluated with the generalized linear mixed-effect model (GLMM), with binomial data distribution. We included, as explanatory variables, week number, altitude, longitude, context (urban/agricultural), and district as random factor (since districts are associated to different surveyors). The GLMM were calculated using package lm4 [38]. The influence of the two sentinel egg mass methods on the parasitoid discovery efficiency was assessed fitting a generalized linear model (GLM) with a quasi-Poisson error distribution, with the type of egg mass (fresh/frozen) and method (cage/direct) as explanatory variables.

## 3. Results

### 3.1. Naturally Laid Egg Masses

Egg masses of ten different stink bug species were collected during the survey, from the first week of June until the second week of October 2019 (Table 1). A total of 581 naturally occurring *H. halys* egg masses were collected, containing both unhatched eggs (18.9%) and parasitized eggs (16.7%). Seven different parasitoid species emerged; the two most abundant species were the indigenous eupelmid *An. bifasciatus* and the exotic scelionid *Tr. mitsukurii*. Several native scelionids (*Tr. cultratus* Mayr, *Tr. belenus* Walker, *Tr. basalis* Wollaston and *Telenomus* spp.) also emerged from *H. halys* eggs. Furthermore, a hyperparasitoid, the pteromalid *Acroclisoides sinicus* (Huang and Liao) emerged from 13 natural laid egg masses, mainly found late in season (September) in urban areas.

**Figure 1:**
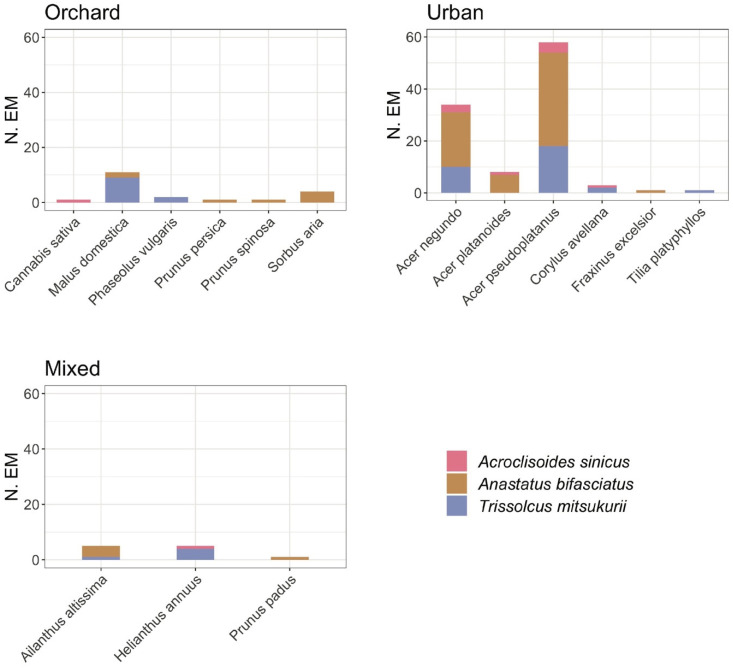
Plant host species and context (orchard, mixed, urban) for the collected *Halyomorpha halys* egg masses parasitized by *Anastatus bifasciatus*, *Trissolcus mitsukurii*, and *Acroclisoides sinicus*; N.E.M (number of egg masses).

*H. halys* egg masses were found on 41 different host plant species (Table S2), mainly on maple trees (*Ac. pseudoplatanus*, *Ac. negundo, Ac. platanoides* L., and *Ac. campestre*) and apple trees (*Malus domestica* (Suckow) Borkh.). The parasitization took place on 29 of these plant species (Table S2). Differences in host plants choice were recorded for the two most abundant parasitoid species and for *Ac. sinicus* (Figure 1). For instance, *Tr. mitsukurii* emergence was recorded on 15 different plant species. Higher parasitism rates for *Tr. mitsukurii* compared to *An. bifasciatus* were registered in orchards, reaching greater parasitism levels on apple trees (*M. domestica*), the most grown crops in the region. On the other hand, the indigenous *An. bifasciatus*, also found on 15 different host plants, showed higher parasitism in urban areas on ornamental plants of public gardens and parking areas, parasitizing egg masses mainly on maple trees.

GLMM supported that the occurrence of *H. halys* parasitization was influenced by the host plant, and that the suitability of plants as host varied with time (Table 2 and Table S3). Three host plants in particular influenced the parasitization: *Ac. pseudoplatanus*, *Ai. altissima*, and *S. aria*. Other environmental variables (i.e., altitude, longitude, and context) were not significant.

### 3.2. Sentinel Egg Masses

Regarding the sentinel egg masses, we exposed 384 egg masses (10,285 eggs) with cages and 121 egg masses directly (3132 eggs). Egg masses directly attached to leaves proved to be more effective than those exposed inside cages (Table 3), both in terms of discovery efficiency (percentage of egg masses with at least a parasitized egg) and exploitation efficiency (percentage of parasitized eggs per egg mass). The probability that an egg mass contained at least a parasitized egg (discovery efficiency) was greater when the sentinel egg masses were exposed directly (quasi-Poisson regression model: Pr (> |t|) < 0.00001), while fresh and frozen eggs did not influence the result (Table S4). Two species of exotic scelionid wasps (*Tr. japonicus* and *Tr. mitsukurii*), and an eupelmid (*An. bifasciatus*) were detected. Egg mass predation was tenfold with direct exposure (3.3%) compared with cages (0.3%), without compromising the survey outcomes.

**Figure 2:**
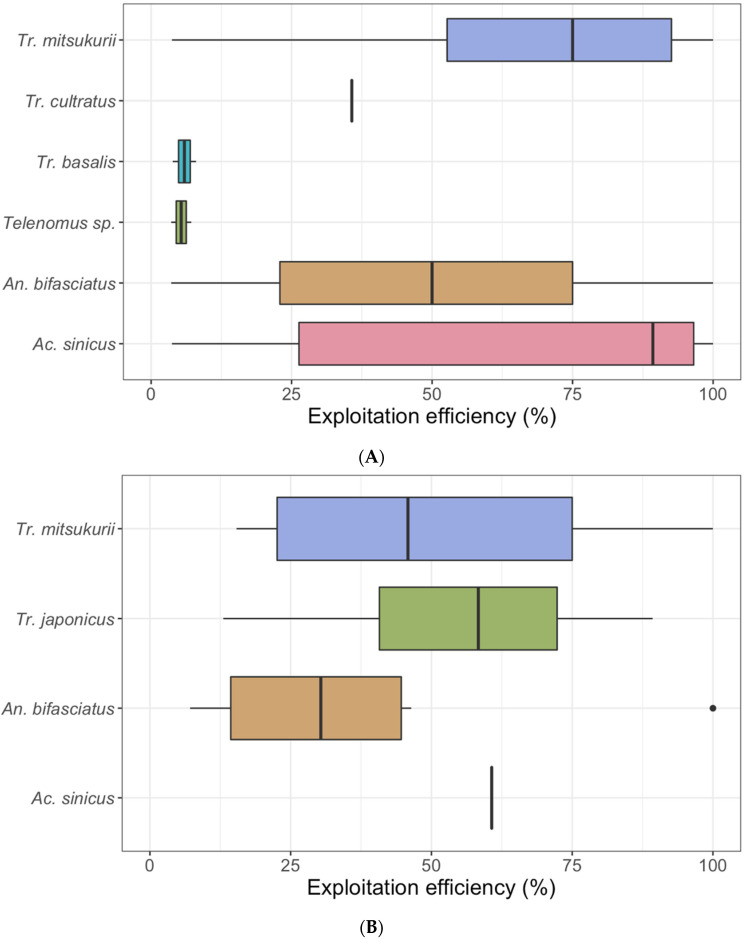
Boxplot of the exploitation efficiency (percentage of parasitized eggs per egg mass) for the emerged parasitoid species, from field collected egg masses (**A**) and sentinel egg masses (**B**).

When comparing the collection of naturally laid egg masses and sentinel egg masses, the exploitation efficacy recorded was higher for the two exotic parasitoids (*Tr. japonicus* and *Tr. mitsukurii*) and for the hyperparasitoid *Ac. sinicus*, compared to the other species (Figure 2. For the naturally collected egg masses, all the species, except *Tr. basalis*, showed an exploitation efficacy per single egg mass above 35%, with peaks of 100% for *Ac. sinicus*, *An. bifasciatus* and *Tr. mitsukurii* (Figure 2). On average, *Tr. japonicus* showed the highest parasitism rate for what concerns sentinel eggs.

**Figure 3:**
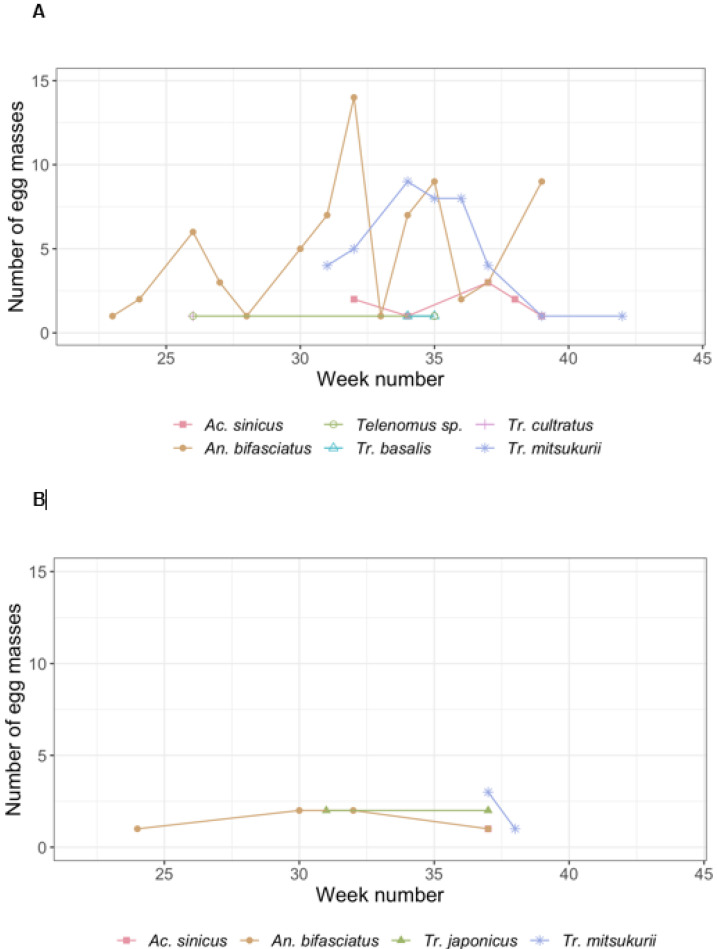
Observed phenology of the emerged parasitoid species from field collected egg masses (**A**) and sentinel egg masses (**B**).

For the observed species’ phenology, the collection of egg masses and the exposure of sentinel egg masses showed similar results (Figure 3), with the native species (*An. bifasciatus*) active from the beginning and throughout the reproductive season of *H. halys*, while the exotic species (*Tr. japonicus* and *Tr. mitsukurii*) were recorded only late in the season (from August), possibly related to the still low densities in the areas where they were introduced.

### 3.3. mtDNA Haplotype Analysis

**Figure 4:**
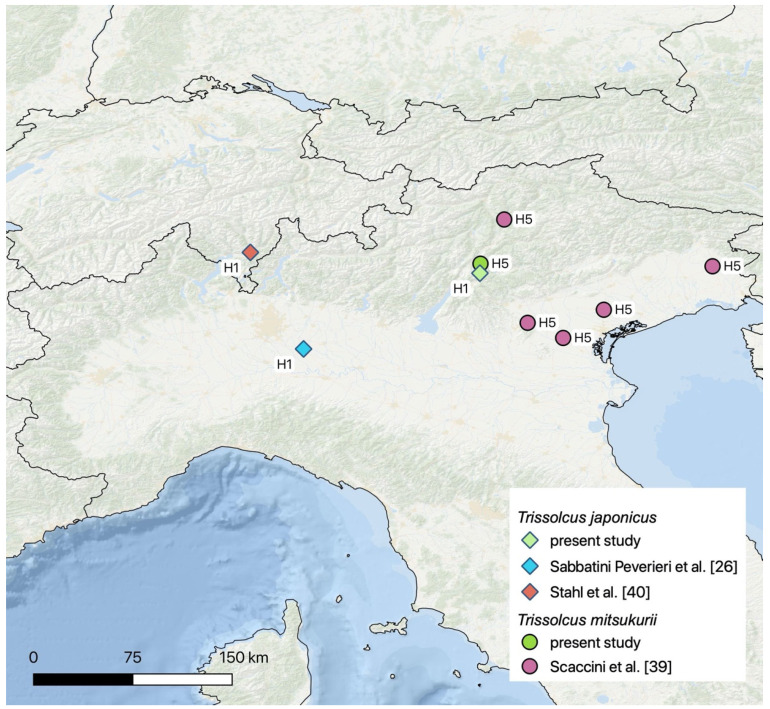
Current distribution of *Trissolcus japonicus* (diamonds) and *Trissolcus mitsukurii* (circles) haplotypes, showing records from the present study and adapted from Sabbatini Peverieri et al. [26], Scaccini et al. [39], and Stahl et al. [16].

All *Tr. mitsukurii* and *Tr. japonicus* specimens targeted for the molecular analysis yielded a ~ 850 bp *CO1* amplicon, and none of the characteristic evidence of numts was present in the sequences obtained. All the sequences generated from this study were deposited in Genbank (Table S1). For both *Tr. mitsukurii* and *Tr. japonicus*, we evidenced a single haplotype across all localities surveyed in the Trentino-Alto Adige Region (Figure 4 and Table S1). The *Tr. mitsukurii* haplotype matched the haplotype H5 evidenced in 2018 in Cordenons (the Friuli-Venezia Giulia region) in Italy [16]. The *Tr. mitsukurii* sequence was also similar to the sequence (Genbank Accession MT345599.1) obtained in Alto Adige in Italy, and recently deposited by Scaccini et al. [39]. The *Tr. japonicus* haplotype matched the haplotype H1, which was found in 2018 in Lodi (Lombardy region) in Italy, in Ticino in Switzerland in 2017, and in Japan [26].

## 4. Discussion

Field surveys combining the collection of naturally laid egg masses and exposure of sentinel egg masses brought new and complementary knowledge on the expansion of both *H. halys*, and its parasitoids. We recorded for the first time, the presence of *Tr. japonicus* in Trentino-Alto Adige, and the rapid expansion of *Tr. mitsukurii*, of which scattered presence was known from the previous year [26]. The direct exposure of sentinel egg masses proved to be more effective than the use of cages, whose structure probably inhibited the entrance of parasitoids. Predation of sentinel egg masses did not represent a limiting factor, with a percentage of incidence lower than what has been recorded in other studies [17]. The fact that *Tr. japonicus* was only detected with sentinel egg masses could be related to its lower density in the area in relation to the other species. Considering that the most recorded species (*An. bifasciatus*, *Ac. sinicus* and *Tr. mitsukurii*) could exploit a broader range of hosts compared to *Tr. japonicus*, the expansion of the latter may have been slower.

Concerning the exploitation efficacy, we obtained similar results with the two methods, with the two exotic parasitoids and the hyperparasitoid showing higher parasitism rates compared to the generalist (*An. bifasciatus)* and other native species. We observed an occasional high percentage of parasitism rate from *An. bifasciatus*, probably resulting from more than one female laying on the same egg mass, suggesting that native parasitoids may be adapting to exploit *H. halys*.

Naturally laid egg masses registered a notable amount of unhatched eggs, which were not further investigated for causes of mortality. Nevertheless, egg mortality could be due to stylet sucking predatory activity, parasitoid induced host-egg abortion, or failed parasitoid development [25,41,42], which underestimate the impact of parasitoids on the host. For instance, the studies conducted by Abram et al. [40] and Konopka et al. [41] showed that both *Telenomus podisi* Ashmead and *Trissolcus euschisti* Ashmead, indigenous scelionids in the US, readily attacked *H. halys* eggs, but were unable to complete their development inside the eggs.

The haplotype similarity observed between our specimens and those from the Region of Friuli-Venezia-Giulia and the Lombardy region [26] strongly suggests that adventive populations of the two egg parasitoids are spreading from wherever they established earlier in Italy. However, as things stand, we cannot completely rule out the hypothesis that adventive populations in Trentino-Alto-Adige originated from the same native area as the other Italian populations and established independently in Italy. Tracing the exact source of the introduction and monitoring the spread of biological control agents depends on the availability of information on the population structure and require analysis of more than one locus [42]. In the present study, we used a new combination of primers that enables the recovery of the *CO1* region that is approximately 200 bp longer than the classical Barcode amplicon [43]. This provided more than enough coverage for the Barcode that is not always obtained when using the universal Folmer primer set. To gain a formal barcode status, the *CO1* sequence must be at least 500 bp long [44]; therefore, this new combination of primers should be considered in the future as a useful supplementary tool for the standard *CO1* barcoding method.

Parasitoid-host selection in Hymenoptera involves several phases, where the location of the host habitat (exploiting plant volatiles and host pheromones) is followed by the actual detection of the host (following its chemical traces) and possibly oviposition [22,45]. The parasitism rates were generally higher for naturally laid eggs than for those observed with sentinel eggs, possibly due to their lack of chemical and vibrational cues. In laboratory conditions, *Tr. japonicus* proved to be attracted by the herbivore-induced plant volatiles (HIPVs) emitted after *H. halys* oviposition [46]. However, the lack of these species-specific signals did not prevent parasitization. Direct exposure of sentinel egg masses on trees where *H. halys* adults and nymphs were present proved to be an effective strategy, more rewarding than the exposure in cages on *Ai. altissima*, even if the latter is one of the few species on which *H. halys* can complete its entire development [47].

Concerning the host species, our results proved that the plant host species affected parasitization and this pattern changed with time. This is in accordance with the fact that the availability of fruits, including wild berries, changes through the season, and thus the attractiveness for *H. halys* and its parasitoids. At short-range, the chemical complexity originating from plant diversity is known to affect the parasitoids’ orientation [48]. In the present study, we observed that the three most common species (*An. bifasciatus*, *Ac. sinicus*, and *Tr. mitsukurii*) were often recorded in different contexts and on different plants. The generalist *An. bifasciatus* and the hyperparasitoid *Ac. sinicus* were more abundant in urban areas (e.g., parking, parks, and gardens, street trees), which are generally characterized by a high diversity of plants and insect hosts. Conversely, the more specialized *Tr. mitsukurii* was often recorded in agricultural areas and, in particular, on *M. domestica*, where rarely other species occurred. The sycamore (*Ac. pseudoplatanus*) was the most common plant host for both *H. halys* and its parasitoids. Since the native rage of sycamore includes most of Europe and the eastern area surrounding the Caspian Sea, our results support that both *H. halys* and its co-evolved parasitoids were able to exploit cues associated to “novel” host plants.

Considering that the exotic parasitoids were found with almost two months of delay compared to native species, we can argue that their peak of activity suffers a time-lag related to their still low densities. The integration of the two monitoring approaches proved to be a successful combination for the detection of scattered populations, providing complementary information—fast detection of emerging species (sentinel egg masses) and ecological characterization (collection of naturally laid eggs).

Agricultural land accounts for the 22% of Trentino and the 40% of Alto Adige (European Rural Development Program data): landscapes with a high degree of intercropping, where vineyards and apple orchards are the predominant crops. Thus, the high number of *H. halys* found on apple orchards was probably influenced by the high availability of the plant. Our results showed that the exotic parasitoids, and in particular *Tr. mitsukurii*, recognized apple orchards as habitat and effectively parasitized *H. halys*.

## 5. Conclusions

Considering that the release of the *Tr. japonicus* for classical biological control programs has just been approved in Italy, these results provide the field evidence that *Trissolcus* species are adapted to our environmental conditions and are able to track their host in their range of introduction. Since our study area offered a high diversity of available host species, our results represent a starting point for evaluating which plant species are more attractive. Understanding which are the key chemical and ecological parameters represents a fundamental step for the development of successful biological control programs [49]. Planting nectar-producing plants has brought increased parasitoid activity with consequent pest reduction [50]. Thus, identifying host plants is fundamental for locating (and creating) hotspots for monitoring *H. halys* native and exotic parasitoids, and for the identification of *Trissolcus* sp. release sites. Further research is required to better understand the role of native and novel host plants for *H. halys* and the exotic *Trissolcus* species in the areas of introduction.

## Figures and Tables

**Table 1:** Collected stink bug species and emerged parasitoids of naturally occurring egg masses (EM: number of egg masses; E: number of eggs; Par: parasitized; Un: unhatched). * Parasitized Eggs include eggs collected after the emergence of parasitoids, according to the characteristics of exit holes.

Stink Bug Species	EM	E	Par. EM	Par. E * (%)	Un. E (%)	Number of Emerged Individuals for Each Species						
						*Acroclisoides sinicus*	*Anastatus bifasciatus*	*Trissolcus basalis*	*Trissolcus belenus*	*Trissolcus cultratus*	*Trissolcus mitsukurii*	*Telenomus* sp.
**Pentatomidae**												
*Halyomorpha halys*	581	15,244	158	2541 (16.7)	2889 (18.9)	201	957	4	15	14	871	3
*Palomena prasina*	42	971	28	508 (52.3)	265 (27.3)		82			200	3	120
*Nezara viridula*	23	2023	7	234 (11.6)	502 (24.8)	20	55	95			6	
*Dolycoris baccarum*	8	206	1	7 (3.4)	21 (10.2)							7
*Pentatoma rufipes*	4	54	2	27 (50)	26 (48.1)					25		
*Carpocoris* sp.	4	54	4	48 (88.9)	6 (11.1)							48
*Rhaphigaster nebulosa*	3	41	1	14 (34.1)	2 (4.9)							14
*Piezodorus lituratus*	1	12	1	2 (16.7)	9 (75)				2			
*Eurydema* sp.	2	24	0	0 (0)	4 (16.7)							
**Coreidae**												
*Gonocerus acuteangulatus*	3	15	1	3 (20)	3 (20)		3					

**Table 2:** Results of the generalized linear mixed-effect models (GLMM) for egg parasitization for *Halyomorpha halys* egg masses, with the logit link function: a) formulation of the best three models; b) significant fixed effects of the best three models. Dev (deviance), Est (estimate), df (df residuals). * *p* < 0.05, ** *p* < 0.01, *** *p* < 0.001.

No	Model Formulation	AIC	BIC	logLik	Dev.	df
(1)	parasitized ~ plant + week n ° + altitude + longitude + context + (1 | district)	523.7	630.7	−236.9	473.7	508
(2)	parasitized ~ plant + week n ° + plant * week n ° + altitude + longitude + context + (1 | district)	518.4	698.1	−217.2	434.4	491
(3)	parasitized ~ plant * week n ° + (1 | district)	517.2	679.8	−220.6	441.2	495
	**Fixed effects:**	**Est.**	**Std. Error**	**z value**	**Pr (>|z|)**	
(1)	(Intercept)	−6.67	1.56	−4.28	<0.001	***
	week n °	0.15	0.03	4.71	<0.001	***
(2)	(Intercept)	−15.32	2.96	−5.18	<0.001	***
	*Acer pseudoplatanus*	11.12	2.93	3.80	<0.001	***
	*Ailanthus altissima*	10.68	4.82	2.22	0.03	*
	*Sorbus aria*	24.86	12.66	1.96	0.05	*
	week n °	0.37	0.08	4.91	<0.001	***
	*Acer pseudoplatanus*: week n °	−0.34	0.09	−3.90	<0.001	***
	*Ailanthus altissima*: week n °	−0.31	0.16	−2.01	0.04	*
(3)	(Intercept)	−12.90	2.55	−5.05	<0.001	***
	*Acer pseudoplatanus*	11.02	2.92	3.77	<0.001	***
	*Ailanthus altissima*	10.42	4.46	2.34	0.02	*
	week n °	0.37	0.08	4.91	<0.001	***
	*Acer pseudoplatanus*: week n °	−0.34	0.09	−3.85	<0.001	***
	*Ailanthus altissima*: week n °	−0.31	0.15	−2.14	0.03	*

**Table 3:** Number of egg masses and mean number of eggs per egg mass used for the sentinel egg mass survey (with cages and direct exposure), showing the discovery efficiency for each approach, the exploitation efficiency (values followed by the same letter were not significantly different according to Kruskal-Wallis H Test), and the collected parasitoid species.

Survey Method						Number of Emerged Individuals for Each Species			
	Egg Mass	Number of Egg Masses	Number of Eggs Per Egg Mass	Discovery Efficiency	Exploitation Efficiency	*Acroclisoides sinicus*	*Anastatus bifasciatus*	*Trissolcus japonicus*	*Trissolcus mitsukurii*
With cages	Fresh	192	26.4 ± 0.15	0%	0.00 ± 0.00	0	0	0	0
	Frozen	192	27.2 ± 0.13	1.60%	0.21 ± 0.13	0	8	3	0
Direct exposure	Fresh	57	25.5 ± 0.37	10.40%	5.68 ± 2.44	0	24	43	10
	Frozen	64	26.7 ± 0.23	9.40%	5.52 ± 2.53	17	21	14	35

## Supplementary Materials

The following are available online at https://www.mdpi.com/2075-4450/11/9/588/s1, Figure S1: Study area map: A. location of the study area in Italy; B. distribution of the exposed sentinel egg masses and the location of the collected egg masses; C. context where the naturally laid egg masses were collected, Table S1: Information on samples of *Trissolcus mitsukurii* and *Trissolcus japonicus* obtained in this study and corresponding CO1 GenBank Accession numbers, Table S2: Collecting period and parasitism rate of naturally occurring *H. halys* eggs according to the host plant. WN (week number), EM (number of egg masses), PEM (number of parasitized egg masses), PE (parasitized eggs), %P (parasitism rate), Table S3: Complete results of the GLMM analysis for egg parasitization for *Halyomorpha halys* egg masses, with logit link function showing the fixed effects of the best three models. Dev (deviance), Est (estimate), df (df residuals), Table S4: Results of the quasi-Poisson regression model for the discovery efficiency of sentinel egg masses exposed directly and with cages.
