# Supplementary material for: Assemblage of the Egg Parasitoids of the Invasive Stink Bug Halyomorpha halys: Insights on Plant Host Associations
Source: Insects. 2020 Sep 1;11(9):588. doi: 10.3390/insects11090588 (PMC7563961; doi:10.3390/insects11090588)
Supplement: Supplementary file 1 [file insects-11-00588-s001.pdf]

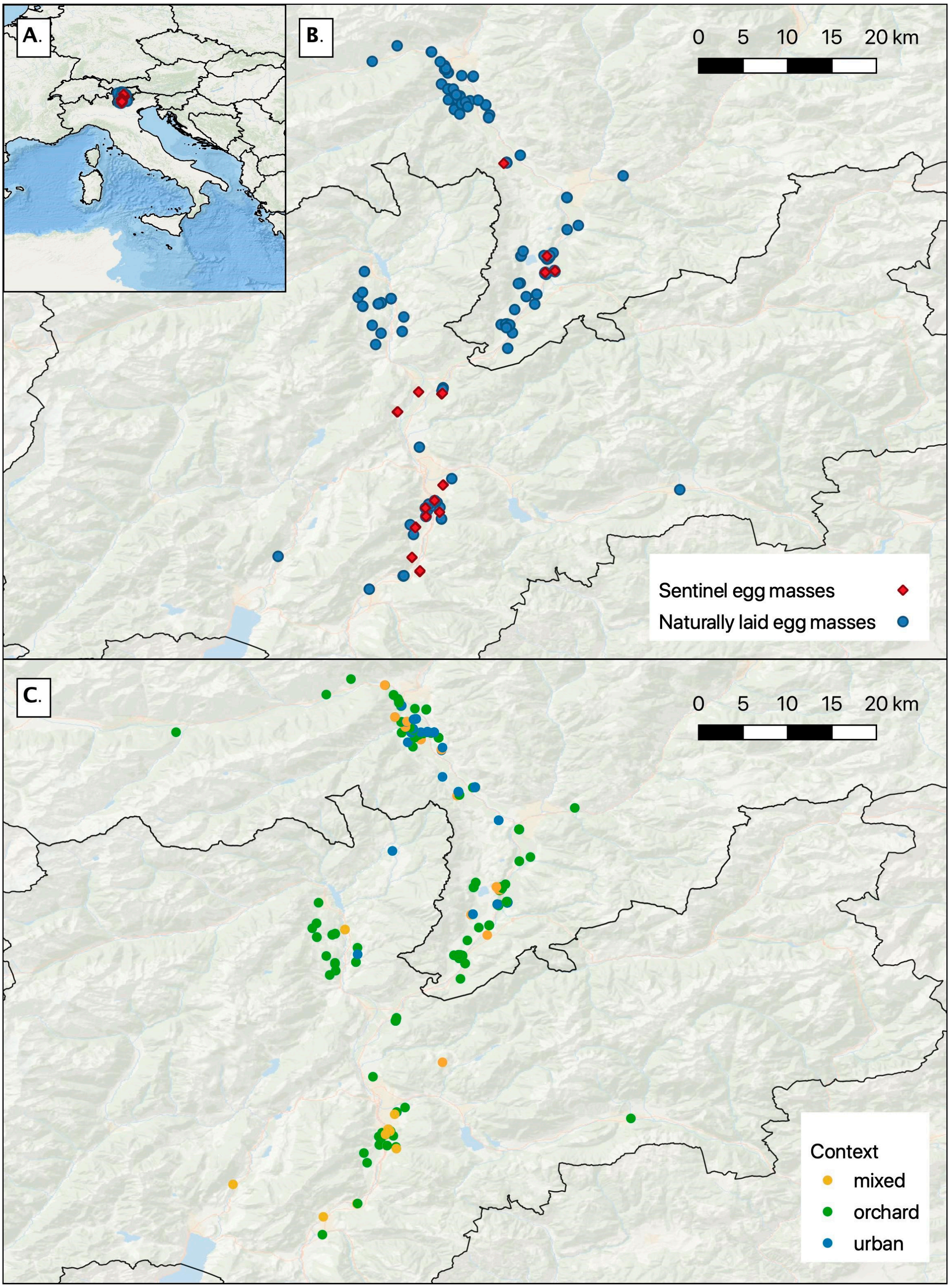

Figure S1. Study area map: **A**. location of the study area in Italy; **B**. distribution of the exposed sentinel egg masses and the location of the collected egg masses; **C**. context where the naturally laid egg masses were collected.

Table S1. Information on samples of *Trissolcus mitsukurii* and *Trissolcus japonicus* obtained in this study and corresponding CO1 GenBank Accession numbers.

Sample ID	Species	Host Plant	Locality in Trentino-Alto Adige in Italy	GPS Coordinates	Collection Date (day/month/year)	*CO1* Genbank Accession Number
TSP361	*Trissolcus mitsukurii*	*Prunus spinosa*	Trento	11,1378914 & 46,0578638	26/07/2019	MT671789
TSP362	*Trissolcus mitsukurii*	*Prunus spinosa*	Trento	11,1378914 & 46,0578638	26/07/2019	MT671790
TSP369	*Trissolcus mitsukurii*	*Diospyros kaki*	Trento	11,1378914 & 46,0578638	12/09/2019	MT671791
TSP370	*Trissolcus mitsukurii*	*Diospyros kaki*	Trento	11,1378914 & 46,0578638	12/09/2019	MT671792
TSP377	*Trissolcus mitsukurii*	*Diospyros kaki*	Trento	11,1378914 & 46,0578638	12/09/2019	MT671793
TSP378	*Trissolcus mitsukurii*	*Diospyros kaki*	Trento	11,1378914 & 46,0578638	12/09/2019	MT671794
TSP383	*Trissolcus mitsukurii*	*Diospyros kaki*	Trento	11,1378914 & 46,0578638	12/09/2019	MT671795
TSP384	*Trissolcus mitsukurii*	*Diospyros kaki*	Trento	11,1378914 & 46,0578638	12/09/2019	MT671796
TSP392	*Trissolcus mitsukurii*	*Malus domestica*	Lidorno	11,1291574 & 46,0301142	14/10/2019	MT671797
TSP393	*Trissolcus mitsukurii*	*Malus domestica*	Lidorno	11,1291574 & 46,0301142	14/10/2019	MT671798
TJ757	*Trissolcus japonicus*	*Acer campestre*	Trento	11,1257637 & 46,0327972	01/08/2019	MT671799
TJ758	*Trissolcus japonicus*	*Acer campestre*	Trento	11,1257637 & 46,0327972	01/08/2019	MT671800
TJ763	*Trissolcus japonicus*	*Prunus persica*	Romagnano	11,1122675 & 46,0222527	12/09/2019	MT671801
TJ764	*Trissolcus japonicus*	*Prunus persica*	Romagnano	11,1122675 & 46,0222527	12/09/2019	MT671802
TJ772	*Trissolcus japonicus*	*Acer campestre*	Trento	11,1257637 & 46,0327972	12/09/2019	MT671803
TJ773	*Trissolcus japonicus*	*Acer campestre*	Trento	11,1257637 & 46,0327972	12/09/2019	MT671804

Table S2. Collecting period and parasitism rate of naturally occurring *H. halys* eggs according to the host plant. WN (week number), EM (number of egg masses), PEM (number of parasitized egg masses), PE (parasitized eggs), %P (parasitism rate).

Host plant	WN	EM	PEM	PE	%P
Acer campestre L.	26–34	4	2	8	7.77
Acer negundo L.	25–39	90	31	515	22.16
Acer platanoides L.	30–32	20	8	113	21.94
Acer pseudoplatanus L.	24–39	151	60	928	23.73
Actinidia deliciosa (A.Chev.) C.F.Liang & A.R.Ferguson	24–36	35	0	0	0
Ailanthus altissima (Mill.) Swingle	23–38	12	4	61	19.61
Cannabis sativa L.	34–37	9	1	3	1.31
Cornus mas L.	24–31	2	0	0	0
Cornus sanguinea L.	34	4	0	0	0
Corylus avellana L.	31–38	6	3	81	53.29
Crataegus monogyna Jacq.	27–31	3	1	9	10.98
Diospyros kaki L.f.	39	1	1	26	92.86
Ficus carica L.	35	2	0	0	0
Fraxinus excelsior L.	27–38	6	1	17	13.08
Hedera helix L.	25–31	4	3	66	58.93
Helianthus annuus L.	31–36	6	4	77	52.03
Juglans regia L.	36	1	1	18	75
Laburnum anagyroides Medik.	31	4	0	0	0
Lactuca sativa L.	35	1	0	0	0
Liquidambar sp. L.	32	1	1	28	100
Malus domestica (Suckow) Borkh.	24–42	120	15	261	8.03
Ocimum basilicum L.	35	1	0	0	0
Paulownia tomentosa (Thunb.) Steud.	24–31	7	0	0	0
Pelargonium zonale (L.) L'Hér.	40	1	0	0	0
Phaseolus vulgaris L.	32	6	2	50	30.3
Prunus armeniaca L.	25–37	3	1	23	30.26
Prunus avium (L.) L.	24–26	12	0	0	0
Prunus cerasus L.	25	1	0	0	0
Prunus domestica L.	32–37	2	2	22	40
Prunus padus L.	26–34	9	1	1	0.42
Prunus persica (L.) Batsch	24–33	7	2	10	5.18
Prunus spinosa L.	24–31	10	1	26	9.85
Punica granatum L.	34	1	1	25	92.59
Sambucus nigra L.	34	1	1	20	71.43
Solanum lycopersicum L.	35	4	1	2	1.9
Sorbus aria (L.) Crantz	24–28	8	4	46	21.2
Syringa sp. L.	34	2	2	33	60
Tilia platyphyllos Scop.	23–34	5	2	32	23.19
Viburnum lantana L.	34	4	1	18	18.95
Vitis vinifera L.	26–40	14	0	0	0
Ziziphus jujuba Mill.	38	1	1	22	78.57
Total	23–42	581	158	2541	16.67

Table S3. Complete results of the GLMM analysis for egg parasitization for *Halyomorpha halys* egg masses, with logit link function showing the fixed effects of the best three models. Dev (deviance), Est (estimate), df (df residuals). * $p < 0.05$, ** $p < 0.01$, *** $p < 0.001$

**Model Formulation**		**AIC**	**BIC**	**LogLik**	**Deviance**	**df.resid**
**parasitized ~ plant + week n° + altitude + longitude + context + (1 | district)**		523.7	630.7	−236.9	473.7	508
Fixed effects:		Estimate	Std.	Error	z value	Pr(>|z|)
(Intercept)		−6.67E + 00	1.56E + 00	−4.283	1.84E − 05	***
Acer	*platanoides*	4.83E − 01	5.21E − 01	0.927	0.3538	
Acer	*pseudoplatanus*	−1.38E − 01	3.17E − 01	−0.437	0.6623	
Actinidia	*deliciosa*	−2.12E + 01	1.35E + 04	−0.002	0.9987	
Ailanthus	*altissima*	2.24E − 01	9.07E − 01	0.246	0.8053	
Cannabis	*sativa*	−1.77E + 00	1.53E + 00	−1.16	0.246	
Corylus	*avellana*	3.15E − 01	8.86E − 01	0.356	0.7221	
Fraxinus	*excelsior*	−5.45E − 01	1.17E + 00	−0.466	0.6412	
Helianthus	*annuus*	1.22E + 00	1.20E + 00	1.011	0.3122	
Malus	*domestica*	−1.76E + 00	1.15E + 00	−1.534	0.125	
Paulownia	*tomentosa*	−2.30E + 01	5.89E + 04	0	0.9997	
Phaseolus	*vulgaris*	−1.50E − 01	1.41E + 00	−0.107	0.9149	
Prunus	*avium*	−2.45E + 01	1.15E + 05	0	0.9998	
Prunus	*padus*	−1.03E + 00	1.18E + 00	−0.877	0.3806	
Prunus	*persica*	−1.05E + 00	1.54E + 00	−0.679	0.4972	
Prunus	*spinosa*	−2.25E − 01	1.55E + 00	−0.145	0.8843	
Sorbus	*aria*	1.87E + 00	1.32E + 00	1.413	0.1576	
Tilia	*platyphyllos*	−2.79E − 01	1.21E + 00	−0.231	0.8175	
Vitis	*vinifera*	−2.32E + 01	4.76E + 04	0	0.9996	
week number		1.54E − 01	3.27E − 02	4.713	2.44E − 06	***
altitude		3.96E − 03	2.13E − 03	1.862	0.0626	.
Longitudine		−2.32E − 04	6.07E − 04	−0.381	0.7028	
context semi-natural		3.37E + 01	1.02E + 07	0	1	
context urban		7.64E − 02	1.05E + 00	0.072	0.9422	
Model formulation		AIC	BIC	logLik	deviance	df.resid
parasitized ~ plant + week n° + plant * week n° + altitude + longitude + context + (1 | district)		518.4	698.1	−217.2	434.4	491
Fixed effects:		Estimate	Std.	Error	z value	Pr(>|z|)
(Intercept)		−1.53E + 01	2.96E + 00	−5.176	2.27E − 07	***
Acer	*platanoides*	−1.08E + 01	1.77E + 01	−0.612	0.54022	
Acer	*pseudoplatanus*	1.11E + 01	2.93E + 00	3.799	0.00015	***
Actinidia	*deliciosa*	−1.63E + 01	1.82E + 06	0	0.99999	
Ailanthus	*altissima*	1.07E + 01	4.82E + 00	2.217	0.0266	*
Cannabis	*sativa*	−6.51E + 02	2.41E + 05	−0.003	0.99785	
Corylus	*avellana*	−2.16E + 02	1.66E + 05	−0.001	0.99896	
Fraxinus	*excelsior*	9.95E + 00	8.00E + 00	1.243	0.21387	
Helianthus	*annuus*	4.96E + 01	2.55E + 01	1.951	0.0511	.
Malus	*domestica*	5.67E + 00	5.70E + 00	0.993	0.32056	
Paulownia	*tomentosa*	−1.11E + 01	9.37E + 05	0	0.99999	
Phaseolus	*vulgaris*	1.39E + 00	1.75E + 00	0.797	0.4256	
Prunus	*avium*	6.14E + 00	2.78E + 06	0	1	
Prunus	*padus*	5.55E + 00	2.31E + 01	0.24	0.80999	
Prunus	*persica*	−4.96E + 02	1.90E + 05	−0.003	0.99791	
Prunus	*spinosa*	2.37E + 02	6.80E + 04	0.003	0.99721	
Sorbus	*aria*	2.49E + 01	1.27E + 01	1.964	0.04952	*
Tilia	*platyphyllos*	−1.80E + 02	8.85E + 04	−0.002	0.99838	
Vitis	*vinifera*	−1.20E + 01	1.42E + 06	0	0.99999	
week number		3.75E − 01	7.63E − 02	4.908	9.21E − 07	***
altitude		4.13E − 03	2.70E − 03	1.529	0.12635	

	Longitudine	−1.75E − 04	7.54E − 04	−0.232	0.81655	
	context semi-natural	3.75E + 01	4.93E + 07	0	1	
	context urban	1.51E + 00	1.45E + 00	1.044	0.2966	
Acer	*platanoides* *week number	3.71E − 01	5.63E − 01	0.659	0.51002	
Acer	*pseudoplatanus* *week number	−3.42E − 01	8.75E − 02	−3.904	9.47E − 05	***
Actinidia	*deliciosa* *week number	−3.41E − 01	6.69E + 04	0	1	
Ailanthus	*altissima* *week number	−3.14E − 01	1.56E − 01	−2.012	0.04426	*
Cannabis	*sativa**week number	1.76E + 01	6.52E + 03	0.003	0.99785	
Corylus	*avellana* *week number	6.37E + 00	4.88E + 03	0.001	0.99896	
Fraxinus	*excelsior* *week number	−3.29E − 01	2.63E − 01	−1.253	0.21025	
Helianthus	*annuus* *week number	−1.46E + 00	7.66E − 01	−1.904	0.05695	.
Malus	*domestica* *week number	−1.86E − 01	1.56E − 01	−1.197	0.23142	
Paulownia	*tomentosa* *week number	−3.75E − 01	3.31E + 04	0	0.99999	
Prunus	*avium* *week number	−1.06E + 00	1.09E + 05	0	0.99999	
Prunus	*padus* *week number	−1.90E − 01	7.45E − 01	−0.255	0.79893	
Prunus	*persica* *week number	1.51E + 01	5.74E + 03	0.003	0.99791	
Prunus	*spinosa* *week number	−9.65E + 00	2.83E + 03	−0.003	0.99728	
Sorbus	*aria* *week number	−7.64E − 01	4.68E − 01	−1.633	0.10257	
Tilia	*platyphyllos* *week number	5.84E + 00	2.84E + 03	0.002	0.99836	
Vitis	*vinifera* *week number	−3.76E − 01	4.66E + 04	0	0.99999	
	Model formulation	AIC	BIC	logLik	deviance	df.resid
	parasitized ~ plant * week n° + (1 | district)	517.2	679.8	-220.6	441.2	495
	Fixed effects:	Estimate	Std.	Error	z value	Pr(>|z|)
(Intercept)		−1.29E + 01	2.55E + 00	−5.054	4.33E − 07	***
Acer	*platanoides*	−1.12E + 01	1.76E + 01	−0.636	0.52448	
Acer	*pseudoplatanus*	1.10E + 01	2.92E + 00	3.771	0.00016	***
Actinidia	*deliciosa*	−1.53E + 01	1.40E + 06	0	0.99999	
Ailanthus	*altissima*	1.04E+01	4.46E + 00	2.336	0.01952	*
Cannabis	*sativa*	-6.53E+02	2.41E + 05	−0.003	0.99784	
Corylus	*avellana*	−2.20E + 02	1.66E + 05	−0.001	0.99895	
Fraxinus	*excelsior*	9.95E + 00	8.00E + 00	1.243	0.21388	
Helianthus	*annuus*	3.61E + 01	1.90E + 01	1.905	0.0568	.
Malus	*domestica*	7.62E + 00	4.93E + 00	1.545	0.12224	
Paulownia	*tomentosa*	−1.65E + 01	7.85E + 06	0	1	
Phaseolus	*vulgaris*	2.15E − 01	9.23E − 01	0.233	0.81576	
Prunus	*avium*	−1.24E + 01	2.78E + 06	0	1	
Prunus	*padus*	8.76E + 00	1.43E + 01	0.612	0.54071	
Prunus	*persica*	−5.13E + 02	1.87E + 05	−0.003	0.99781	
Prunus	*spinosa*	2.34E + 02	6.91E + 04	0.003	0.9973	
Sorbus	*aria*	2.31E + 01	1.26E + 01	1.836	0.06635	.
Tilia	*platyphyllos*	−1.82E + 02	9.00E + 04	−0.002	0.99839	
Vitis	*vinifera*	−1.34E + 01	1.28E + 06	0	0.99999	
	week number	3.75E − 01	7.63E − 02	4.908	9.21E − 07	***
Acer	*platanoides* *week number	3.85E − 01	5.62E − 01	0.684	0.49368	
Acer	*pseudoplatanus* *week number	−3.36E − 01	8.73E − 02	−3.848	0.00012	***
Actinidia	*deliciosa* *week number	−3.59E − 01	5.13E + 04	0	0.99999	
Ailanthus	*altissima* *week number	−3.13E − 01	1.46E − 01	−2.139	0.03245	*
Cannabis	*sativa**week number	1.76E + 01	6.52E + 03	0.003	0.99785	
Corylus	*avellana* *week number	6.48E + 00	4.88E + 03	0.001	0.99894	
Fraxinus	*excelsior* *week number	−3.29E − 01	2.63E − 01	−1.253	0.21027	
Helianthus	*annuus* *week number	−1.06E + 00	5.77E − 01	−1.842	0.06548	.
Malus	*domestica* *week number	−2.79E − 01	1.44E − 01	−1.936	0.05284	.
Paulownia	*tomentosa* *week number	−3.75E − 01	2.77E + 05	0	1	
Prunus	*avium* *week number	−3.70E − 01	1.09E + 05	0	1	
Prunus	*padus* *week number	−3.08E − 01	4.60E − 01	−0.669	0.5035	
Prunus	*persica* *week number	1.55E + 01	5.66E + 03	0.003	0.99781	
Prunus	*spinosa* *week number	−9.58E + 00	2.88E + 03	−0.003	0.99735	

Sorbus	*aria* *week number	−7.58E − 01	4.68E − 01	−1.619	0.10549
Tilia	*platyphyllos* *week number	5.91E + 00	2.91E + 03	0.002	0.99838
Vitis	*vinifera* *week number	−3.76E − 01	4.21E + 04	0	0.99999

Table S4. Results of the quasi-Poisson regression model for the discovery efficiency of sentinel egg masses exposed directly and with cages. * $p < 0.05$, ** $p < 0.01$, *** $p < 0.001$

Coefficients:	Estimate	Std. Error	*T* Value	Pr(>|t|)	
(Intercept)	−5.0236	0.616	−8.155	2.84E−15	***
Method: direct exposure	2.5427	0.6065	4.193	3.26E−05	***
Egg: fresh/frozen	0.316	0.4952	0.638	0.524	
